# Upadacitinib as a treatment for co-existent allergic contact dermatitis and psoriasis

**DOI:** 10.1016/j.jdcr.2023.11.023

**Published:** 2023-12-07

**Authors:** Peter Yi Ch’en, Miriam Al-Saedy, Eingun James Song

**Affiliations:** a Albert Einstein College of Medicine, Bronx, New York; b Elson S. Floyd College of Medicine, Spokane, Washington; c Frontier Dermatology, Mill Creek, Washington

**Keywords:** allergic contact dermatitis, biologic therapies, JAK-STAT pathway, psoriasis, upadacitinib monotherapy

## Introduction

Atopic dermatitis (AD) and psoriasis have divergent and antagonistic immune mechanisms, explaining the rarity of the presence of both diseases in the same individual. AD has been primarily shown to be a helper T cell (Th)2-driven disease, whereas psoriasis is caused by Th17 immune activation.^1^ Biologic therapies that selectively target the Th2 and Th17 immune pathways have been transformative in treating these conditions. However, rare cases of the development of eczema in patients receiving psoriasis biologics or development of de novo psoriasiform dermatitis in patients receiving dupilumab (Dupixent), which is an interleukin 4 (IL-4) receptor subunit antagonist that blocks Th2-mediated cytokines, have been reported in the literature and referred to as “phenotypic switching” by some.^2^^,^^3^

More commonly, allergic contact dermatitis (ACD) can develop in patients with psoriasis, but treatment remains challenging because our current biologic therapies may be too targeted to treat both entities. The Janus kinase (JAK)-STAT pathway is involved in cytokine signaling for a multitude of inflammatory disorders, including AD, ACD, and psoriatic arthritis.^4^ As such, JAK inhibitors have the potential to treat co-existent psoriatic and atopic disease. This article reports 2 cases of patients referred for psoriasis in whom concomitant eczematous dermatitis developed, which we were able to successfully treat with upadacitinib (Rinvoq) monotherapy.

## Case report

### Case 1

A 50-year-old man with a diagnosis of psoriasis from his 2 previous dermatologists presented to our clinic for a third opinion. His treatment history included topical steroids, secukinumab (Cosentyx), tildrakizumab (Ilumya/Ilumetri), and, most recently, ixekizumab (Taltz). On initial examination, his palms and soles were diffusely red and hyperkeratotic with deep fissures of the fingertips and irregular pitting of his fingernails. The patient reported previous involvement of his scalp and glans penis. Because he was only 4 weeks into ixekizumab therapy when he presented to us, he agreed to a full 3-month trial while adding oral methotrexate 15 mg/wk.

At his 2-month follow-up, the patient reported a new, itchy, red rash involving his suprapubic skin and flanks. Two shave biopsies from his suprapubic skin and back showed spongiosis with abundant eosinophils. The working diagnosis was an IL-17-induced eczematous dermatitis versus superimposed ACD. The patient declined patch testing and elected to be treated with intramuscular triamcinolone 80 mg while increasing methotrexate to 20 mg/wk.

**Fig 1:**
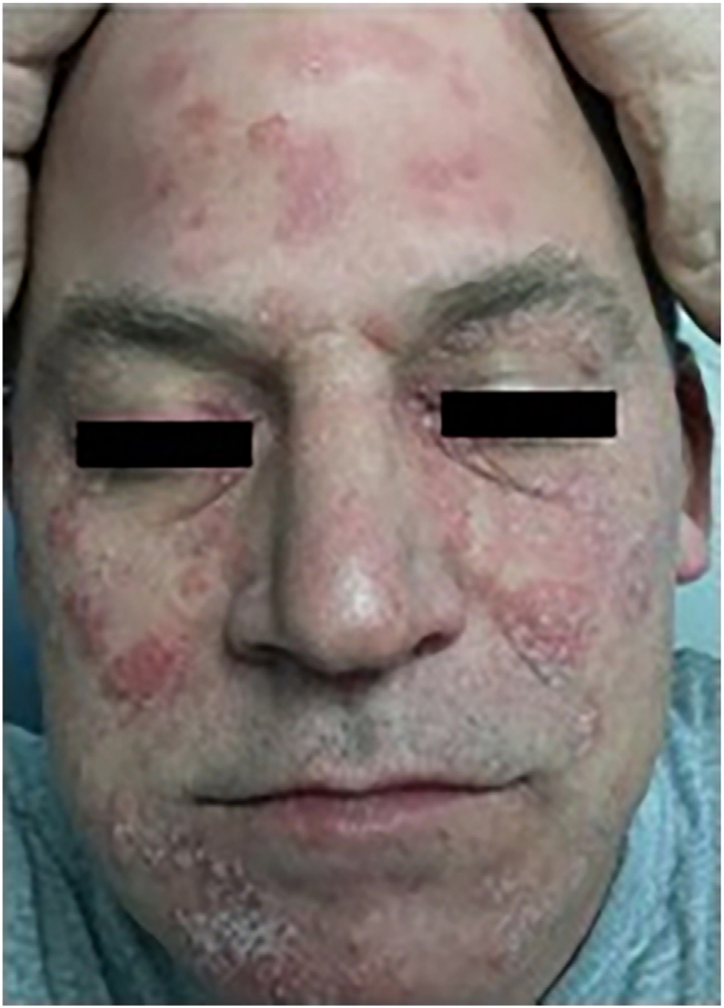
At 9-month follow-up of patient 1, classic psoriasiform plaques were seen on the scalp, face, elbows, knees, thighs, intergluteal cleft, and glans penis.

At his 3-month follow-up, the patient reported that his rash was worsening, spreading now to his scalp and hands. Ixekizumab and methotrexate were discontinued, and dupilumab was initiated. Three months into treatment, patient was at least 90% improved with some residual skin thickening of his palms and peeling of his fingertips without a relapse of his psoriasis. Nine months after starting dupilumab, the patient in whom classic psoriasiform plaques had developed on the scalp, face, elbows, knees, thighs, intergluteal cleft, and glans penis, affecting approximately 5% of his body surface area (Fig 1). A shave biopsy was performed on his left flank, which demonstrated changes consistent with psoriasis.

**Fig 2:**
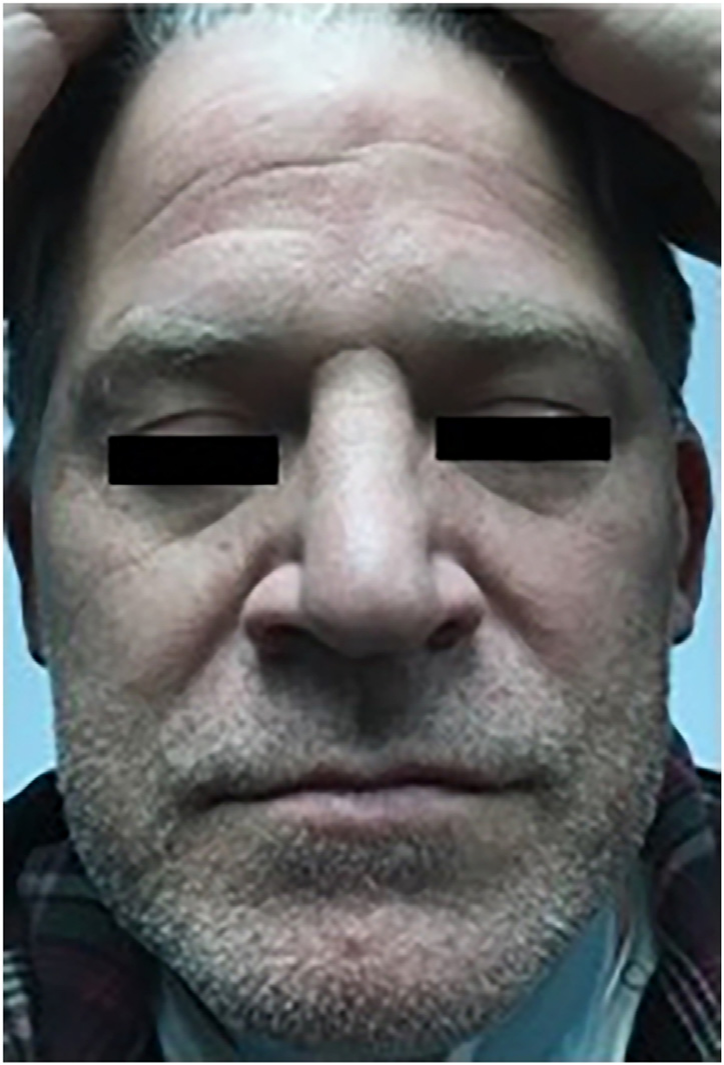
Patient 1 was completely clear within 1 week of upadacitinib 15 mg daily.

Eventual patch testing revealed numerous contact allergens, including a 2+ reaction to Balsam of Peru and 1+ reaction to fragrance mix. Notably, the patient’s wife sold scented cinnamon candles and essential oils that she diffused throughout the house. The patient reported difficulty avoiding all fragrances because of the nature of his wife’s work. Dupilumab was discontinued, and upadacitinib 15 mg daily was started. At his 1-week follow-up, his psoriasiform had completely cleared (Fig 2). As of today’s writing, the patient has been receiving upadacitinib 15 mg daily for 24 months and both his psoriasis and eczematous dermatitis have remained clear.

### Case 2

**Fig 3:**
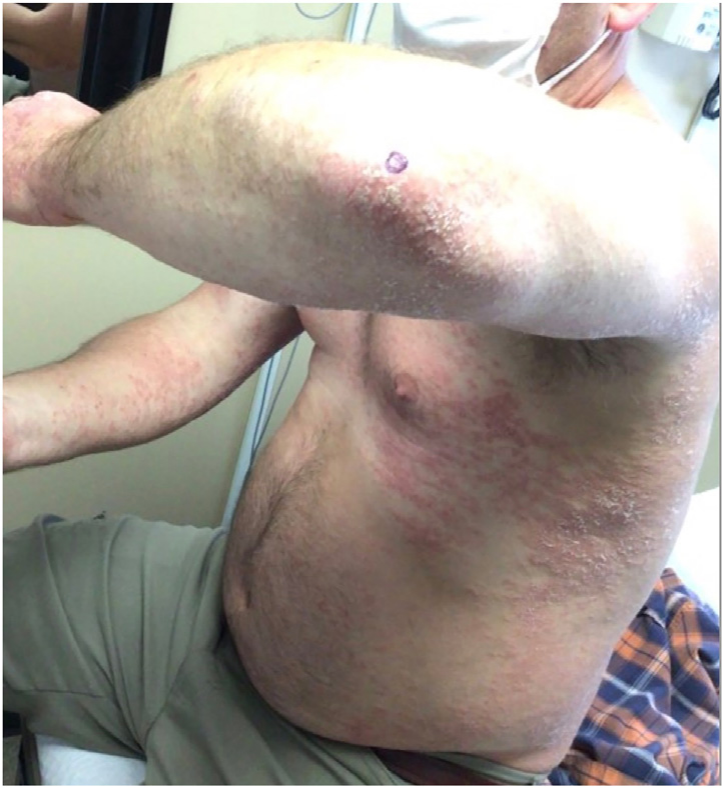
Patient 2 presented with widespread psoriasis that was confirmed by histopathology.

A 45-year-old man presented to our clinic with widespread psoriasiform dermatitis involving 30% of the body surface area (Fig 3). A shave biopsy was performed, confirming the diagnosis of psoriasis. The patient started receiveing oral methotrexate with escalation to 20 mg/wk, but persistent elevated liver enzymes prompted switching to certolizumab (Cimzia) 400 mg every 2 weeks. At 2 months, he achieved a body surface area of <1%. However, at his 6-month follow-up, intensely pruritic, deep-seated vesicles developed on both palms with widespread eczematous dermatitis on the trunk (Fig 3). Shave biopsies revealed spongiotic dermatitis with eosinophils. The differential diagnosis was tumor necrosis factor-induced eczematous dermatitis versus superimposed ACD.

Certolizumab was discontinued, and the patient started receiving dupilumab 600 mg subcutaneously at week 0 and 300 mg every 2 weeks thereafter. At his 3-month follow-up, his entire torso had cleared, but he had persistent eczematous patches and plaques on the hands and forearm without a return of his psoriasis. Patch testing revealed a 1+ reaction to fragrance mix and lavender. Interestingly, the patient’s wife had a lavender business. Despite protective clothing and avoidance, the patient continued to break out on his hands and forearms.

Dupilumab was discontinued, and the patient started receiving upadacitinib 15 mg daily. At his 2-month follow-up, the patient was completely clear. However, he had an episode of shingles of his T6 dermatome that resolved with valacyclovir. As of today’s writing, the patient has remained clear with upadacitinib 15 mg daily for 12 months without a return of his psoriasis.

## Discussion

Biologic therapies that selectively target the Th2 and Th17 immune pathways have been transformative in treating inflammatory diseases. However, rare cases of the development of eczema in patients receiving psoriasis biologics or the development of de novo psoriasiform dermatitis in patients receiving dupilumab have been reported in the literature and referred to by some as phenotypic switching.^2^^,^^3^^,^^5^ In the author’s experience, some of these cases of phenotypic switching were in fact psoriasis with co-existent ACD, as we believe our 2 cases demonstrated.

Dupilumab has shown inconsistent results in treating ACD, whereas several case reports of oral JAK inhibitors successfully treating ACD have been published.^6^ Tofacitinib (Xeljanz) was shown to treat refractory parthenium-induced airborne ACD, whereas abrocitinib (Cibinqo) was able to treat a case of airborne ACD to compositae in a patient refractory to topical steroids and dupilumab.^7^^,^^8^

Given the importance of JAK signaling in immune-mediated inflammatory disorders, JAK inhibitors have the potential to treat co-existent inflammatory disorders. Upadacitinib is approved for both moderate-to-severe AD and psoriatic arthritis. In the pivotal SELECT-PsA 1 study, the psoriasis area severity index 75/90/100 was 62/50/34, respectively at 16 weeks with the 30 mg dose.^9^

Although both patients had relevant contact allergens, we cannot prove that this was the cause of the secondary eczematous dermatitis. It is conceivable that both of these patients had a phenotypic switch from the use of targeted biologic therapy, although the distribution and history were more suggestive of secondary ACD. A case of a phenotypic switch from eczema to psoriasis on upadacitinib has also been recently reported; therefore, there may be a subset of patients in whom upadacitinib may not work.^10^ Nonetheless, we have demonstrated that upadacitinib effectively treats psoriasis with co-existent ACD in 2 individuals.

## Conflicts of interest

None disclosed.

## References

[1] Guttman-Yassky E., Krueger J.G. (2017). Atopic dermatitis and psoriasis: two different immune diseases or one spectrum?. Curr Opin Immunol.

[2] Al-Janabi A., Foulkes A.C., Mason K., Smith C.H., Griffiths C.E.M., Warren R.B. (2020). Phenotypic switch to eczema in patients receiving biologics for plaque psoriasis: a systematic review. J Eur Acad Dermatol Venereol.

[3] Brumfiel C.M., Patel M.H., Zirwas M.J. (2022). Development of psoriasis during treatment with dupilumab: a systematic review. J Am Acad Dermatol.

[4] Suzuki K., Meguro K., Nakagomi D., Nakajima H. (2017). Roles of alternatively activated M2 macrophages in allergic contact dermatitis. Allergol Int.

[5] Megna M., Caiazzo G., Parisi M. (2022). Eczematous drug eruption in patients with psoriasis under anti-interleukin-17A: does interleukin-22 play a key role?. Clin Exp Dermatol.

[6] Hendricks A.J., Yosipovitch G., Shi V.Y. (2021). Dupilumab use in dermatologic conditions beyond atopic dermatitis - a systematic review. J Dermatolog Treat.

[7] Muddebihal A., Sardana K., Sinha S., Kumar D. (2023). Tofacitinib in refractory Parthenium-induced airborne allergic contact dermatitis. Contact Dermatitis.

[8] Baltazar D., Shinamoto S.R., Hamann C.P., Hamann D., Hamann C.R. (2022). Occupational airborne allergic contact dermatitis to invasive Compositae species treated with abrocitinib: a case report. Contact Dermatitis.

[9] McInnes I.B., Anderson J.K., Magrey M. (2021). Trial of upadacitinib and adalimumab for psoriatic arthritis. N Engl J Med.

[10] Ferrucci S.M., Buffon S., Marzano A.V., Maronese C.A. (2022). Phenotypic switch from atopic dermatitis to psoriasis during treatment with upadacitinib. Clin Exp Dermatol.

